# Non-conventional forms of HLA-B27 are expressed in spondyloarthritis joints and gut tissue

**DOI:** 10.1016/j.jaut.2016.03.009

**Published:** 2016-06

**Authors:** Oliwia Rysnik, Kirsty McHugh, Leonie van Duivenvoorde, Melissa van Tok, Giuliana Guggino, Joel Taurog, Simon Kollnberger, Francesco Ciccia, Dominique Baeten, Paul Bowness

**Affiliations:** a Nuffield Department of Orthopaedics, Rheumatology and Musculoskeletal Science, University of Oxford, Oxford, UK; b Amsterdam Rheumatology and Immunology Center, Department of Clinical Immunology and Rheumatology, Department of Experimental Immunology Academic Medical Center, University of Amsterdam, Amsterdam, The Netherlands; c Dipartimento di Biopatologia e Biotecnologie Mediche e Forensi, Dipartimento Biomedico di Medicina Interna e Specialistica, Sezione di Reumatologia, Università di Palermo, Italy; d Department of Internal Medicine, Rheumatic Diseases Division, University of Texas Southwestern Medical Center, Dallas, USA; e Cardiff Institute of Infection & Immunity, Henry Wellcome Building, Heath Park, Cardiff CF14 4XN, UK

**Keywords:** HLA-B27, HLA class I free-heavy chains, Spondyloarthropathies, HLA-B27 transgenic rat model

## Abstract

**Objectives:**

Human leukocyte antigen (HLA)-B27 (B27) is the strongest genetic factor associated with development of Ankylosing Spondylitis and other spondyloarthropathies (SpA), yet the role it plays in disease pathogenesis remains unclear. We investigated the expression of potentially pathogenic non-conventional heavy chain forms (NC) of B27 in synovial and intestinal tissues obtained from SpA patients. We also determined the presence of NC-B27 in joints, lymphoid and gastrointestinal tissue from B27 transgenic (TG^1^) rats with *M.tuberculosis*-induced SpA.

**Methods:**

Expression of NC-B27 in human SpA joints and gut and in (21-3 × 283-2)F_1_ HLA-B27/Huβ2m rat tissue was determined by immunohistochemistry, flow cytometry and confocal microscopy analysis using HC10 and HD6 antibodies.

**Results:**

Both HC10- and HD6-reactive HLA molecules were present in synovial tissue from SpA patients. Both NC-B27 and KIR3DL2, a ligand for NC-B27, were expressed in inflamed terminal ileal tissues in patients with early SpA. Infiltrating cells in inflamed joint tissues isolated from B27 TG^1^ rats expressed high levels of NC-B27. NC-B27 were also expressed in joint-resident cells from ankle and tail joints of B27 TG^1^ rats prior to clinical arthritis. The expression of NC-B27 on B27 TG^1^ rat CD11b/c^+^, CD8α^+^, cells from spleens and LNs increased with animal age and disease progression.

**Conclusions:**

Non-conventional HLA class 1 molecules are expressed on resident and infiltrating cells in both synovial and GI tissues in human SpA. NC-B27 expression in joints and lymphoid tissues from B27 TG^1^ rats prior to the onset of arthritis is consistent with the hypothesis that they play a pathogenic role in SpA.

## Highlights

• HLA-B27 predisposes to Ankylosing Spondylitis and related Spondyloarthritis (SpA).
• Non-conventional HLA-B27 free heavy chains (NC-B27) bind innate immune receptors.
• NC HLA class I molecules are expressed in joint and gut tissues in human and rat SpA.
• B27 transgenic rats express NC-B27 prior to arthritis onset.
• These data support a pathogenic role for non-conventional B27 in SpA.

## 1 Introduction

The spondyloarthropathies (SpA) are a group of closely related chronic inflammatory diseases that share genetic, clinical and pathophysiological features. The very strong association of the human leukocyte antigen HLA-B27 (B27) with SpA was discovered more than four decades ago, yet the role B27 plays in disease pathogenesis remains unclear. We have previously described the ability of HLA-B27 to form beta 2 microglobulin-free homodimers [1]. These and other non-conventional forms of B27 (NC-B27), including free heavy chain forms (FHC), can be expressed both intracellularly [2], and at the cell surface [1,3]. Increased expression of cell surface FHC has been demonstrated on SpA patient-derived monocytes and peripheral blood mononuclear cells (PBMCs) [4], [5], [6]. Recent GWAS studies have identified IL23R and other genes involved in generation of “type 17” immune responses as important in Ankylosing Spondylitis (AS) [7], [8], [9], [10], [11]. Furthermore, several clinical and animal studies have suggested the importance of IL23R/IL-17 axis in pathogenesis of SpA, including AS and PsA [5,[12], [13], [14], [15], [16], [17], [18], [19]. We have previously shown that cell surface-expressed NC-B27 can interact with innate immune receptors including KIR3DL2 expressed on NK and CD4^+^ T cells [20], [21], [22], [23], [24], and that this interaction can drive Th17 immune responses [5,15].

These findings led us to ask two important questions: a) are these NC-B27 forms expressed in the inflamed target tissues in SpA? and b) is this expression a consequence of inflammation or does it precede inflammation?

Here we describe expression of NC-B27 in joint and gut tissue in both human SpA and in (21-3 × 283-2) F_1_ HLA-B27/Huβ2m B27 transgenic rats including those with *M. tuberculosis*-induced arthritis and spondylitis.

## 2 Materials and methods

### 2.1 Patients

Human synovial tissue samples were obtained with informed consent and appropriate ethical permission, from 4 B27^+^ SpA patients, including 1 with Ankylosing Spondylitis (AS) fulfilling the New York classification criteria [25], and 10 patients with Rheumatoid Arthritis (RA) fulfilling the EULAR/ACR criteria [26]. Gastrointestinal tissue samples were obtained, with informed consent and appropriate ethical permission, from 10 AS B27^+^ patients and 10 healthy B27^−^ individuals undergoing ileocolonoscopy for diagnostic purposes but without clinical evidence of underlying disease.

### 2.2 Rat-derived cells and tissues

B27 transgenic (TG) rats first generated by Hammer and colleagues spontaneously develop inflammatory gut and joint disease [27], and express B27 homodimers on their dendritic cells [28], and NC-B27 molecules on splenocytes [29]. However NC-B27 expression has not been studied using the more recent (21-3 × 283-2) F_1_ HLA-B27/Huβ2m TG rat model, in which additional human β2m is introduced. We hereafter term this model B27 TG^1^. A higher proportion of these B27 TG^1^ male rats spontaneously develop arthritis (∼70%, 4–6 months of age) and spondylitis (30–50%, 7–9 months of age) without symptoms of gut inflammation [30], [31], [32]. Early and coordinated onset of these SpA-like disease manifestations can be triggered by immunization with low doses of heat-inactivated *M. tuberculosis* (hereafter referred to as “*M.tb*-induced arthritis and spondylitis”) [33,34]. Splenocytes, lymph node cells (LNs), ankle, tail joints and GI tissues were isolated from B27 TG^1^ rats with spontaneous or induced SpA at age 4–15 weeks. For *M.tb*-induced arthritis and spondylitis [33], 6 week-old B27 TG^1^ rats were immunized with 30–45 μg of heat-inactivated *M.tb* in incomplete Freund's adjuvant. (120-4 × 283-2)F_1_ HLA-B7/Huβ2m TG (B7 TG) and Lewis wild type (WT) animals ± 200 μg of heat-inactivated *M.tb* in IFA (adjuvant-induced arthritis, AIA model) were used as controls. All animals were bred and housed at the animal facility of the AMC, University of Amsterdam, Netherlands. All animal procedures were carried out in compliance with Institutional Standards for Human Care and Use of Laboratory Animals.

### 2.3 Antibodies

The HC10 antibody stains many or all heavy chain forms (but not beta-2-microglobulin-associated conventional forms) of most human HLA-B and some HLA-A alleles, but does not cross react with rat MHC [35]. The HD6 antibody was raised against B27 homodimers using a fully human FAb antibody library (kindly provided by Dynax, MA, USA) as previously described [5], and is more specific for heavy chain forms of HLA-B27. HD6r (same specificity as HD6 but with rat IgG1 Fc region) was used for some stains. The M-K323-12B11, anti-KIR3DL2 mAb was a kind gift of Innate Pharma, France. W6/32 and ME1 recognize beta-2 microglobulin-associated class 1 molecules, as used in previous studies [29].

### 2.4 Immunohistochemistry of human and rat tissue samples

Human SpA and RA, and rat paraffin-embedded synovial tissue samples were prepared as previously described [32,^36^,37]. Paraffin-embedded tissue sections were blocked using Peroxidase Blocking Reagent (EnVision™, Dako), than incubated with PBS/1%FBS/10% goat serum and subsequently stained overnight with HC10 or HD6 primary mAb. HC10-stained sections were incubated with HRP-labeled anti-mouse IgG (EnVision™, Dako). HD6-stained sections were incubated with biotinylated goat anti-mouse IgG1 (Southern Biotech) followed by streptavidin-HRP (Dako). Tissue sections were than incubated with AEC^+^ substrate-chromogen (EnVision™, Dako) and counterstained using Mayer's hematoxylin. Slides were visualized and scanned using AperioCS2 Scanner and analyzed using Aperio ImageScope software (Leica Biosystems, UK).

Frozen GI tissue sections from B27^+^ AS patients and healthy controls were blocked before incubation with primary antibodies: HD6r, M-K323-12B11 or isotype control. Tissues were next incubated with biotinylated secondary antibody followed by incubation with streptavidin-HRP, then developed with diaminobenzidine and counterstained with hematoxylin. The number of positive cells was determined by evaluating reactive cells on microphotographs taken from three randomly selected high-power microscopic fields under a Leica DM2000 optical microscope.

Double staining for HD6r and CD3 or CD68 were performed on frozen ileal sections. Sections were treated with FITC-, RR- or Cy-5-conjugated anti-mouse or anti-rabbit antibodies (Invitrogen) plus RNase and counterstained using DAPI (Life Technologies). Antibody staining was analyzed by immunofluorescence confocal microscopy.

### 2.5 Flow cytometry

Splenocytes and LNs were freshly isolated and immediately stained as described previously [29]. Cells were incubated in blocking buffer, and then stained with primary antibody (HC10, HD6, ME1 or IgG1/IgG2a), followed by incubation with secondary goat anti-mouse antibody (Alexa Fluor 647, Invitrogen). Subsequently, cells were stained for the phenotypic surface markers: CD4 and CD8α or CD45R and MHCII, or CD11b/c. Dead cells were excluded using fixable viability dye eFluor^®^780 (eBioscience). Flow cytometric analysis was performed with BD FACS Canto and data were analyzed using FlowJo Software (TreeStar). Staining was performed in triplicates. Error bars were calculated based on SD mean of the values if 3 ≥ animals per group. P values were determined using nonparametric Mann-Whitney test.

## 3 Results

### 3.1 Non-conventional HLA class I molecules are detected on synovial cells in the intimal and sublining layers in B27^+^ SpA, but not in RA tissues

**Fig. 1:**
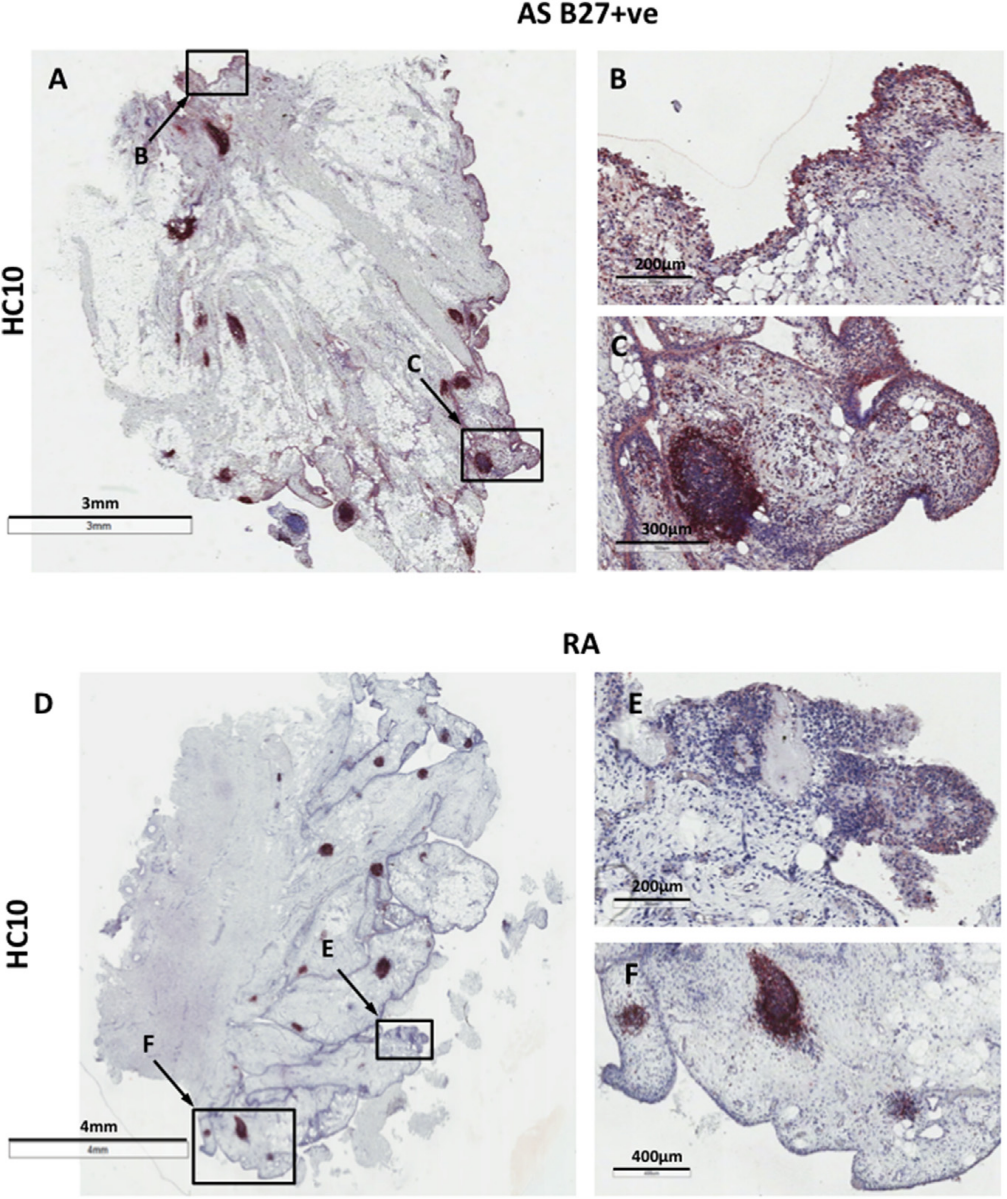
**HC10-expressing cells are present in the intimal and sublining layers of synovial tissue from B27**^**+**^**AS patients but not from RA patients. (**A–C) HC10 staining in paraffin-embedded synovial tissue sections from inflamed B27^+^ AS patient peripheral joint: (A) 0.7X, (B) HC10 + ve cells in the synovial tissue intimal and sublining (8X), (C) lymphoid cell infiltrates/follicles (10X). (D–F) HC10 staining in inflamed synovial tissue from RA patient with peripheral arthritis is present only on cell infiltrates in lymphoid follicles, but is absent in the intimal and sublining layers of synovial tissue: (D) 0.3X, (E) cells in the synovial tissue intimal and sublining (8X), (F) HC10 staining in lymphoid cell infiltrates/follicles (10X). All images were captured at 20X objective lens using AperioScanner and analyzed in ImageScope. Representative of 4 B27^+^ SpA patients and 10 RA patients.

We first wished to determine whether non-conventional HLA I molecules were expressed in the inflamed peripheral joints from SpA patients. Fig. 1 shows that HC10 consistently stained the intimal and sublining layer cells in synovial tissues from B27^+^ SpA but not RA patients. HC10 staining was also detected on mononuclear cells found in lymphoid follicles in synovial tissue in both SpA and RA (Fig. 1, C and F). Of note we observed similar patterns of HC10 and HD6 staining in both frozen and paraffin-fixed synovial SpA tissues (see Fig. 1 in Ref. [38]). HD6 staining was detected in intimal and sublining layers and on cells in the lymphoid follicles in paraffin-embedded tissues from SpA patients, although background staining on both SpA and RA synovial tissues was seen (see Fig. 1 in Ref. [38] and data not shown). Due to the tissue preparation and IHC staining procedures used in this study, we did not observe staining with ME1 and W6/32 antibodies (data not shown).

### 3.2 Gut tissue cells from HLA-B27-positive AS patients express both higher levels of non-conventional HLA class I molecules and the natural killer receptor KIR3DL2

**Fig. 2:**
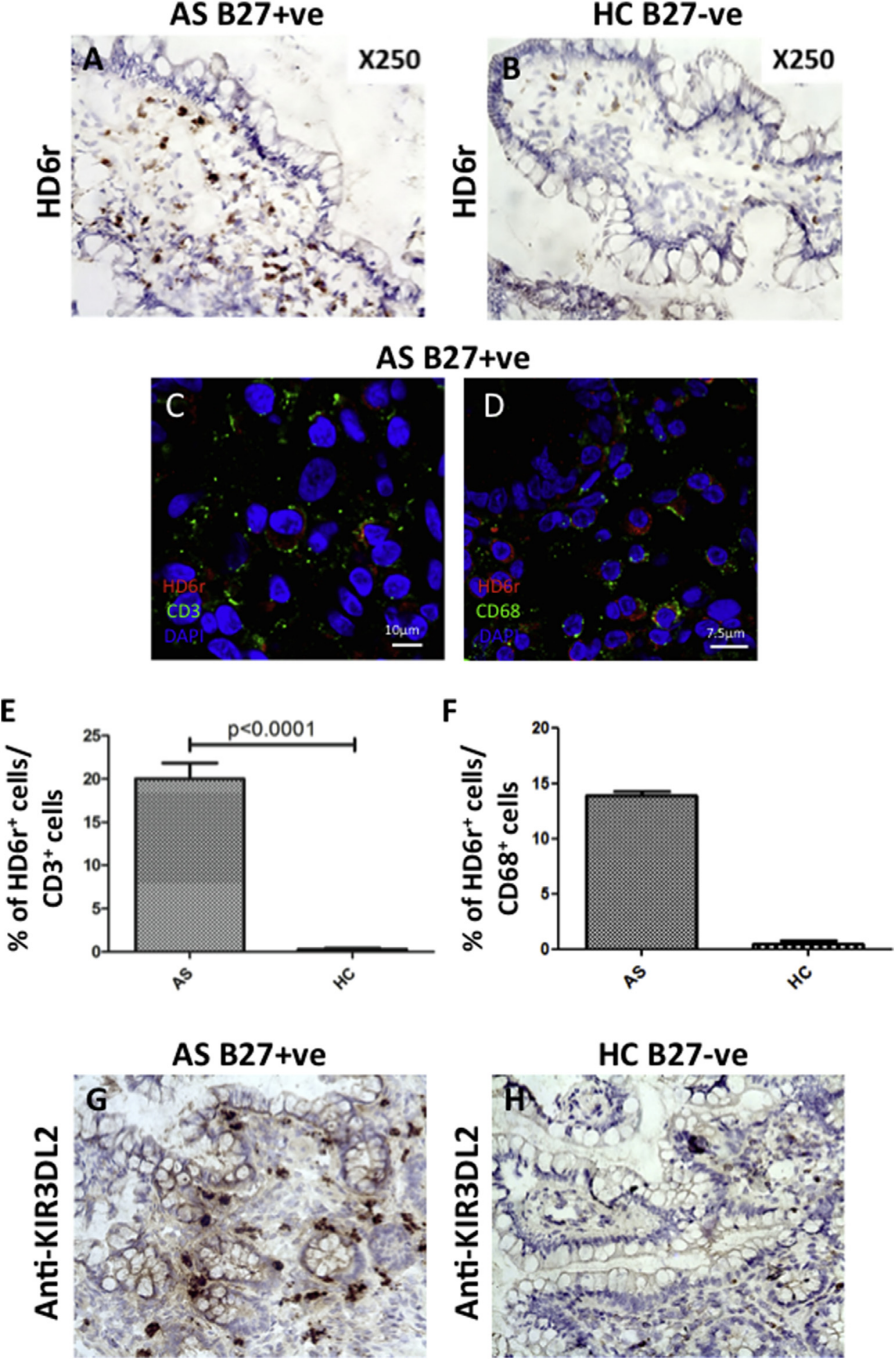
**HD6**^**+**^**and KIR3DL2**^**+**^**cells are present in B27**^**+**^**AS gastrointestinal tissue**. (A, B) HD6(r) staining of frozen gastrointestinal tissue sections from (A) a B27^+^ AS patient (n = 10) and (B) a healthy B27^−^ control (n = 10). (C, D) Immunofluorescence double staining of frozen gastrointestinal tissue with (C) HD6r (red) and CD3 (green), or (D) HD6r (red) and CD68 (green) from a B27^+^ AS patient. Nuclear staining DAPI (blue). (E, F) quantification of (E) HD6r^+^/CD3^+^ and (F) HD6r^+^/CD3^+^ double positive cells in frozen gastrointestinal tissue from B27^+^ AS patient. (G, H) anti-KIR3DL2(M-K323-12B11, Innate Pharma, France) staining of frozen gastrointestinal tissue sections from (G) a B27^+^ AS patient and (H) a healthy B27^−^ control. (For interpretation of the references to colour in this figure legend, the reader is referred to the web version of this article.)

To extend our finding that non-conventional class 1 molecules are expressed in synovial tissue, we next looked for expression in another target tissue in SpA, the gut mucosa. We used the HD6r mAb to determine whether NC-B27 molecules are present in human (GI) tissues from B27^+^ AS patients with subclinical inflammation. HD6 staining was observed in GI tissue sections from B27^+^ AS patients independently of the degree of gut inflammation, but not in tissues from B27^−^ healthy controls (Fig. 2, A vs B). The majority of HD6 staining was present on infiltrating mononuclear cells in the lamina propria. Further immunofluorescence analysis of frozen sections showed that HD6 staining was seen on both CD3^+^ and CD68^+^ cells (Fig. 2, C–F). We also studied mononuclear cell expression of the natural killer receptor KIR3DL2, which strongly binds NC-B27 [20]. Fig. 2G shows the presence of abundant mononuclear cells expressing KIR3DL2 in ileal tissue from a B27^+^ AS patient. This was not seen in healthy control tissue (Fig. 2H). Thus both NC-B27 and KIR3DL2, a key ligand for NC-B27, are expressed in GI tissue from AS patients with subclinical inflammation.

### 3.3 NC-B27 molecules are highly expressed on cells in the synovium, at bone remodeling sites in inflamed joints and in the gut of B27 TG^1^ rats with *M.tb*-induced arthritis and spondylitis

**Fig. 3:**
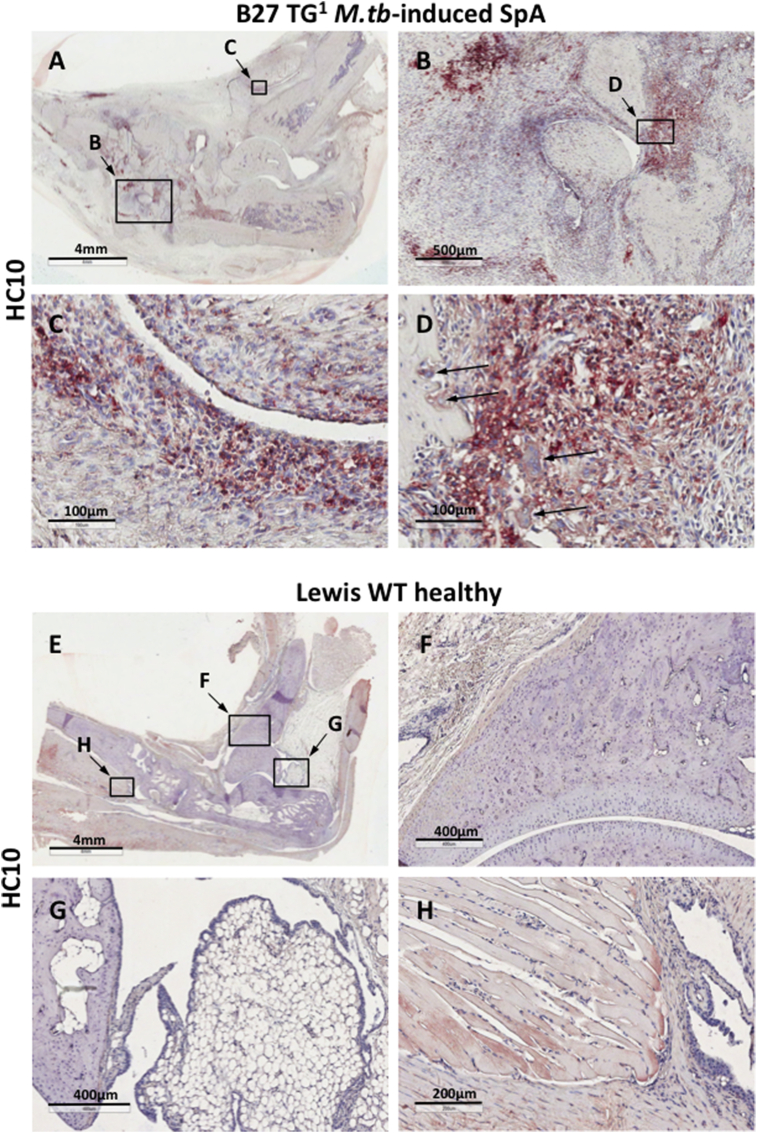
**HC10 staining is present on cells in the synovium and at bone remodeling sites in inflamed ankle joints from B27 TG**^**1**^**but not control Lewis rats. (**A–D) HC10 staining in paraffin-embedded ankle joint from *M.tb*-induced SpA: (A) 0.6X; (B) 4X; (C) 20X view of intimal and sublining layers of the synovial tissue; D) 20X view showing staining of mononuclear cells and multinucleated osteoclast-like cells (arrows) in bone remodeling sites. (E–H) HC10 staining of healthy control Lewis WT rat ankle joint: (E) 0.5X; (F) bone and chondrocyte cartilage sites (5X); (G) bone and attached synovial and adipose tissue (6X); (H) synovial tissue in the muscle attachment area (10X). All images were captured at 20X objective lens using AperioScanner and analyzed in ImageScope. Representative of 5 animals.

We next asked if NC forms are expressed in the joints of B27 transgenic rats with arthritis and spondylitis. Fig. 3 shows markedly increased expression of NC-B27 in inflamed ankle joints from B27 TG^1^ rats (12–15 weeks old) with *M.tb*-induced arthritis and spondylitis (compared with Lewis WT rats). HC10 staining was present on infiltrating mononuclear cells (Fig. 3B), on mononuclear cells in the intimal and sublining layers of synovial tissue (Fig. 3C), and on cells at bone remodeling sites (Fig. 3D). HC10 staining was also observed on multinucleated (osteoclast-like) cells at bone remodeling sites and on bone marrow mononuclear and multinuclear cells (Fig. 3D arrows). Comparable HC10 staining was observed in axial joints from B27 TG^1^ rats with *M.tb*-induced SpA, particularly in cell infiltrates at the junction between the vertebrae, connective tissue and annulus fibrosus (see Fig. 2 in Ref. [38]). We did not observe HC10 staining in ankle or tail joints from healthy Lewis WT rats (Fig. 3E-H and Fig. 2E and F in Ref. [38]), or in ankle joints from Lewis WT rats with adjuvant-induced arthritis (AIA) (see Fig. 3 in Ref. [38]).

**Fig. 4:**
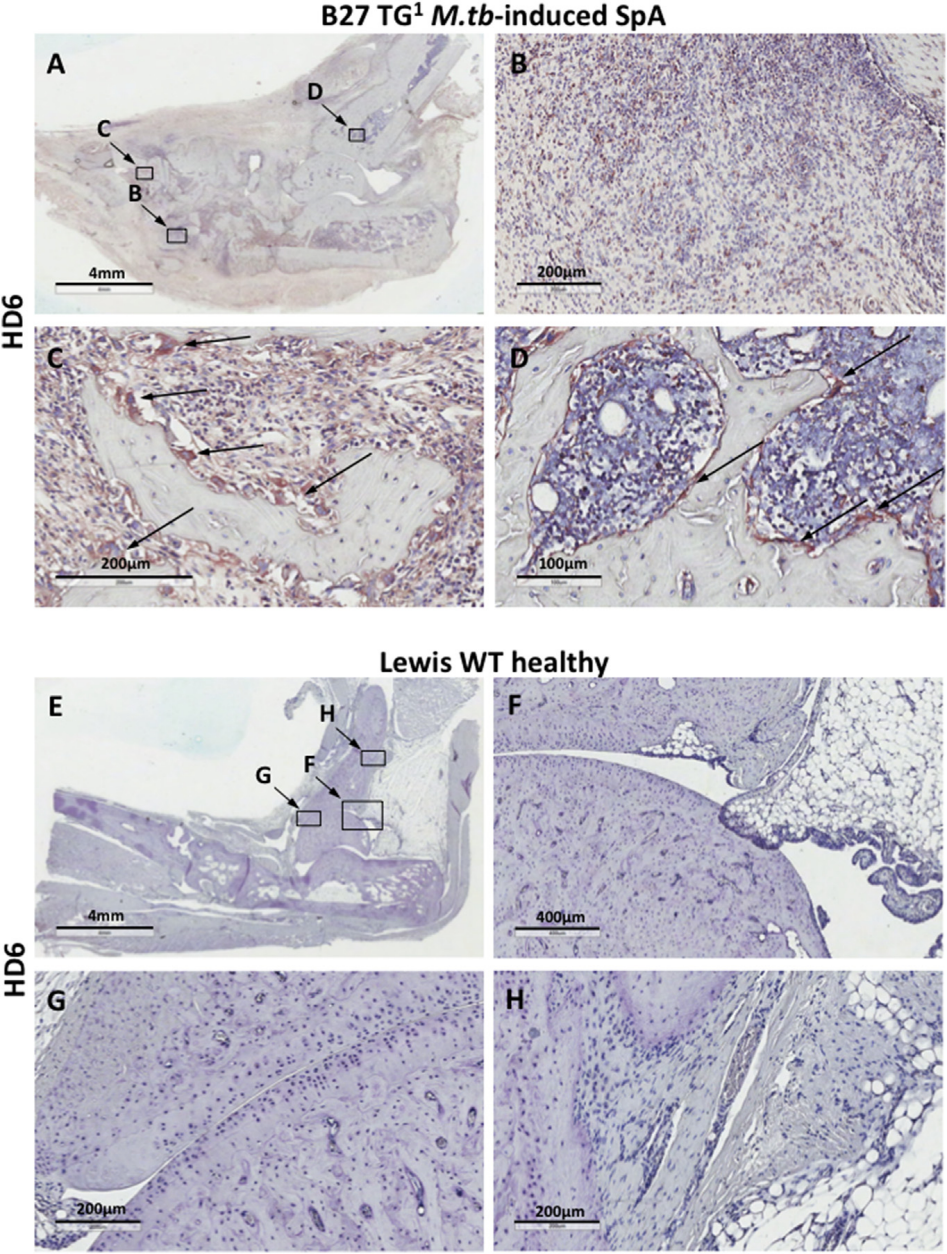
**HD6 staining is present on cells in the synovium, at bone remodeling sites and in the bone marrow in ankle joints from B27 TG**^**1**^**but not control Lewis rats. (**A–D) HD6 staining in paraffin-embedded ankle joint tissue section from diseased B27 TG^1^ rat. (A) 0.6X; (B) 10X; (C) increased HD6 staining on multinucleated osteoclast-like cells in bone remodeling site (20X); (D) HC10^+^ cells in bone marrow (20X). (E–H) HD6 staining was not detected in control Lewis WT rats with no disease: (E) 0.6X; F) and (G) bone and chondrocyte cartilage sites with surrounding synovium (5X and 10X); (H) bone and connective and adipose tissue near the bone area. All images were captured at 20X objective lens using AperioScanner and analyzed in ImageScope. Representative of 5 animals.

We further investigated expression of NC-B27 in the joints of B27 TG^1^ rats with *M.tb*-induced arthritis and spondylitis, using the HD6 mAb generated against B27 homodimers [Bibr bib5][Bibr bib39],. As with HC10, HD6 staining was observed on infiltrating mononuclear cells in ankle joints (Fig. 4A and B), and on mononuclear cells at bone remodeling sites (Fig. 4C). Notably, multinucleated osteoclast-like cells present at bone remodeling sites (Fig. 4C, arrows) and mononuclear cells in bone marrow (Fig. 4D, arrows) stained strongly with HD6. Similar HD6 staining was observed in axial joints (Data not shown). No HD6 staining was seen for ankle (Fig. 4E–H) or tail joints from healthy Lewis WT animals (Data not shown) or for ankle joints from Lewis WT animals with AIA (see Fig. 3E–H in Ref. [38]). Tissue sections from B27 TG^1^ rats with *M.tb*-induced arthritis and spondylitis did not stain with IgG1 isotype control (see Fig. 4 in Ref. [38]). Thus these data show that NC-B27 forms are expressed in the inflamed joints of rats with arthritis and spondylitis.

Since B27 transgenic rats [27] develop both gut and joint inflammation, we next looked for evidence of NC-B27 expression in gut tissues from B27 TG^1^ rats and control B7 TG and Lewis WT rats. HC10 staining was detectable on mononuclear cells in small intestinal Peyer's patches, in lymphoid follicles and in the lamina propria of all transgenic animals. Staining levels were higher for B27 TG^1^ rats with *M.tb*-induced arthritis and spondylitis compared to those without *M.tb* or healthy B7 TG animals (see Fig. 5 in Ref. [38]). Specific HC10 staining was also seen in the colon (see Fig. 6 in Ref. [38]). HD6 stained small and large bowel tissues of B27 TG^1^ rats with *M.tb*-induced arthritis and spondylitis (see Fig. 7 in Ref. [38]). Thus NC-B27 are expressed in gut tissue in B27 TG^1^ rats.

### 3.4 NC-B27 expression on CD11b/c^+^ and CD8α^+^ cells from B27 TG^1^ rats increases with disease onset. NC-B27 expression can be detected in rat joints at a stage preceding overt arthritis

**Fig. 5:**
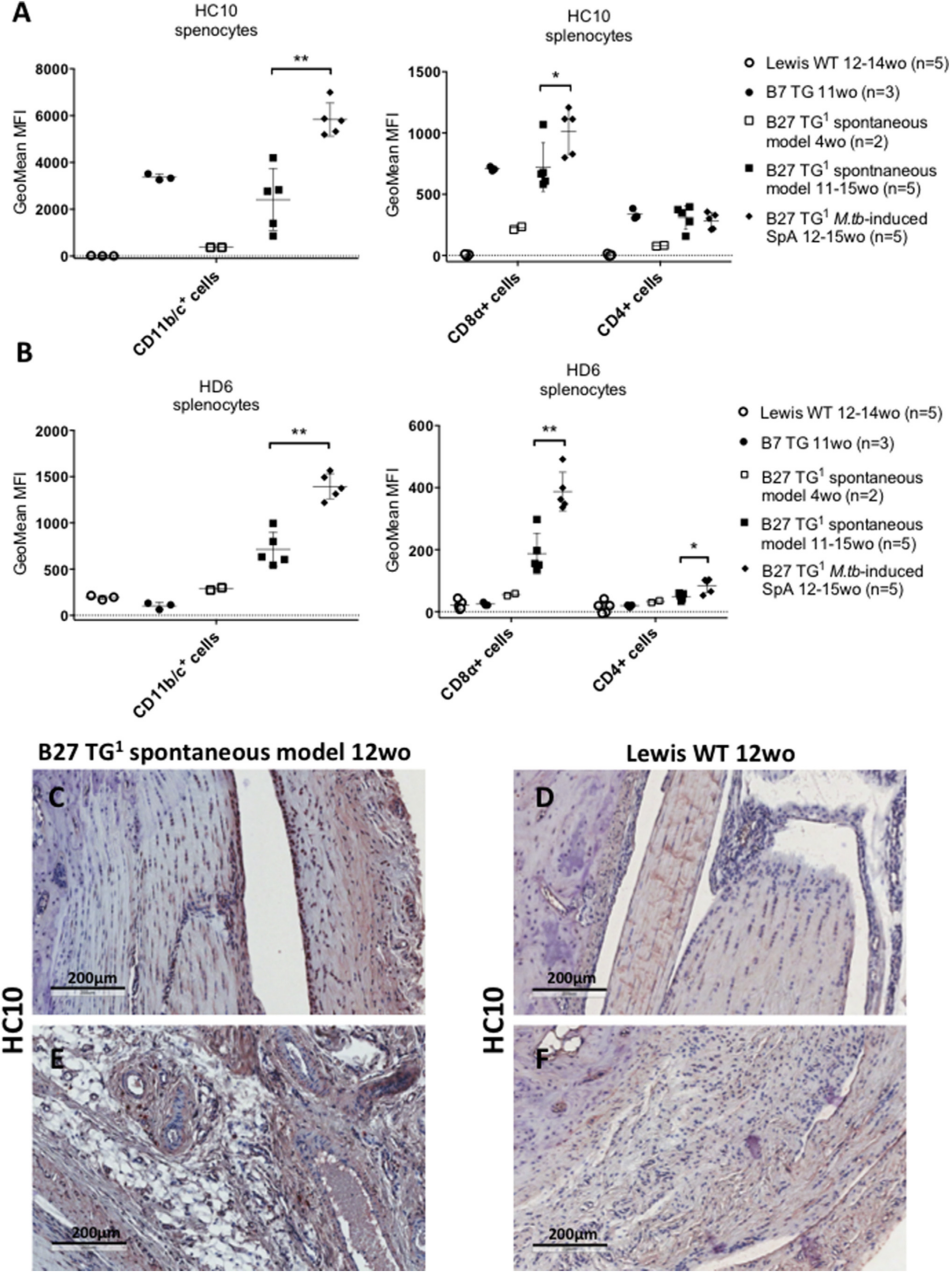
**NC-B27 expression on CD8α**^**+**^**and CD11b/c**^**+**^**cells from B27 TG**^**1**^**rats increases with disease onset and is detectable in joints before clinical manifestations of arthritis**. (A) HC10 staining and (B) HD6 staining on CD11b/c^+^, CD8α^+^ and CD4^+^ splenic cells isolated from B27 TG^1^ rats; spontaneous model (4 wo, open squares, n = 2 and 11-15wo, closed squares, n = 5), and *M.tb*-induced rats (11–15 wo, closed diamonds, n = 5). B7 TG (11 wo, closed circles, n = 3) and Lewis WT (12–14 wo, open circles, n = 5) rat splenocytes were used as controls. Staining was performed in triplicates. Error bars were calculated based on SD mean of the values if 3≥ animals per group. P values were determined using nonparametric Mann-Whitney test HC10 staining of ankle joints from (C, D) 12 wo B27 TG^1^ animals (spontaneous model) prior to disease onset and (E, F) healthy ankle joints from aged matched Lewis WT rats. IHC staining is a representative of 5 animals.

We also studied expression of NC-B27 in spleens and lymph nodes taken from B27 TG^1^ animals prior to disease onset (spontaneous model) and from animals with *M.tb*-induced arthritis and spondylitis. Expression of both HC10- and HD6-reactive molecules was significantly higher on splenic CD11b/c^+^ monocytic cells (monocytes, dendritic cells or macrophages) (Fig. 5A, left) and splenic CD8α^+^ cells (Fig. 5A, right) from the *M.tb*-induced animals compared with age-matched B27 TG^1^ (spontaneous model). CD4^+^ cells (Fig. 5A, right) also stained with HC10 and HD6, however the intensity of staining was lower compared with CD8α^+^ and CD11b/c^+^ cells. We did not observe any significant difference in the staining between age-matched B27 TG^1^ animals with and without clinical symptoms. CD45^+^/MHCII^+^ cells expressed very low levels of NC-B27 molecules (see Fig. 8 in Ref. [38]). Similar results were observed with cells isolated from B27 TG^1^ lymph nodes ± *M.tb* (see Fig. 9A in Ref. [38]), excepting that the CD11b/c^+^ cell population was absent (see Fig. 5 and 10J in Ref. [38]). We also investigated HC10 and HD6 staining of splenocytes taken from 8 to 9 weeks old B27 TG^1^ animals with and without *M.tb*-induced SpA before the appearance of clinical manifestations. HC10 was not significantly altered in splenic CD11b/c^+^, CD8α^+^ or CD4^+^ cells, or on cell populations from LNs (see Fig. 9B–D in Ref. [38]). However, we observed an increase in HD6 staining on splenic CD4^+^ cells after *M.tb* treatment (see Fig. 9C in Ref. [38]). No HC10 or HD6 staining was observed in splenic and LN cells from age-matched Lewis WT rats (data not shown). Splenic and LN cells from age-matched B7 TG rats stained with HC10, but not HD6, to a similar degree compared with B27 TG^1^ animals (spontaneous model) (Fig. 5A and B and see Fig. 9A in Ref. [38]).

Lastly we asked if NC-B27 expression precedes or is a consequence of inflammation? Fig. 5C–F shows that HC10 staining could be detected in the joints of B27 TG rats at 12 weeks, sacrificed shortly before the expected onset of overt arthritis. These data indicate that increased NC-B27 expression precedes the onset of clinical inflammation in B27 TG^1^ rats.

## 4 Discussion

Here we show that NC-B27 molecules are expressed in the synovium and small bowel of B27^+^ SpA patients and in the joints, gut tissues and lymphoid organs of B27 TG^1^ rats with *M.tb*-induced arthritis and spondylitis. We observed HC10 staining of intimal and sublining layers of synovial tissue from both human SpA patients and B27 TG^1^ rats, as well as of inflammatory cell infiltrates/lymphoid follicles. Whilst HC10 staining was also observed in synovial tissue from RA patients, staining of synovial intimal and sublining layers was specific to B27^+^ SpA patients. Our data suggest that resident or infiltrating cells in the intimal and sublining layers of the synovium could play a role in the arthritis of SpA patients. These cells include T cells and macrophages [40]. Of note in the GI tissue sections from B27^+^ AS patients both CD3^+^ cells (which could be TCR γδ or αβ cells) and CD68^+^ cells stained with HD6. Although B27 TG^1^ rats do not develop gastrointestinal inflammation, we observed increased intensity of HC10 staining in GI tissues from B27 TG^1^ rats with axial and peripheral arthritis compared with animals before the onset of clinical manifestations. However, it is yet unclear whether the changes in the expression of NC-B27 in rat GI tissues are a cause or a consequence of joint inflammation. The effects of diet and microbiome on NC-B27 expression may be also be significant and undoubtedly also merit detailed investigation.

NC-B27 molecules were expressed by cells in ankle and axial joints and in peripheral lymphoid organs from B27 TG^1^ animals. NC-B27 expression was also detected in the joints of unimmunized animals shortly before the expected onset of arthritis. We also observed significant increases in NC-B27 expression on different leukocyte populations with increasing age in (21-3 × 283-2) F_1_ (B27 TG^1^) animals (±*M.tb)*. Similar changes have been reported in an earlier B27 TG rat model, in which animals develop severe gastrointestinal inflammation but arthritis is present at lower frequency [29]. Treatment of these B27 TG rats with a novel antibody to B27 dimers (HD5) has recently been shown to reduce cell surface B27 dimer expression, to reduce numbers of TNF- and IL-17-producing CD4 T cells, and to enhance weight gain [19], lending support to a pathological role for NC-B27.

Here we measured NC-B27 expression using two monoclonal antibodies with overlapping specificities, HC10 and HD6. HC10 recognizes β2m-free heavy chain forms of most HLA-B and some HLA-C alleles [35]. HD6 has a greater specificity for B27 homodimers [5,39]. Both HC10- and HD6-reactive forms of NC-B27 have been shown to bind to the killer immunoglobulin-like receptor KIR3DL2 [20], [23]. Binding of NC-B27 to KIR3DL2 promotes the expansion of NK and T cells [15,^20^,41]. Cell surface HC10-reactive molecules are found on all cell types expressing HLA class I. Our findings, consistent with several other studies, suggest that their expression in SpA-like disease is increased [4,^6^,^42^,43]. Due to the tissue preparation and IHC staining procedures used in this study, we did not observe any staining with ME1 and W6/32 antibodies. We also cannot be certain to what extent observed NC-B27 staining is located at the cell surface. Whilst it is possible that some NC-B27 staining results from sample preparation, we observed similar patterns of HC10 and HD6 staining on frozen (as compared to paraffin-fixed) synovial SpA tissues, indicating that a significant proportion of NC-B27 are present at the cell surface.

Although expression of the KIR3DL2 receptor has been shown previously on synovial NK and T cells, here we show for the first time KIR3DL2 expression in the gut of AS patients. Although the present study lacks mechanistic work from human tissue, we have previously shown that NC-B27 interactions with KIR3DL2 expressed on CD4^+^ T cells promote Th17 immune responses in vitro [15]. Notably, increased Th17 responses have been observed in B27 TG rats, with reduction by an antibody to NC-B27, suggesting similar pathogenesis [19].

Based upon our findings we propose a model in which cell surface expression of NC-B27 is a key factor in initiating or more likely exacerbating joint and gut inflammation in SpA. Our data do not conclusively differentiate between expression by resident cells and infiltrating cells, although one possibility is that *M.tb* may accelerate the progression of arthritis and spondylitis in B27 TG^1^ rats - via the activation and/or recruitment of cell populations infiltrating the axial and peripheral joints. Direct interaction of these mononuclear cells with NC-B27-expressing cells present in joints may then exacerbate immune responses. Our results are also consistent with models in which intracellular expression of NC-B27 in gut or joint cells initiates inflammation through ER stress or autophagy [2], or depletion of together with dendritic cell abnormalities [44], as intra-cellular and cell surface NC-B27 expression can occur together [3,45]. Notably aberrant class 1 expression may also drive inflammation in related diseases including psoriasis and Behcet's disease, and this has led to the concept of “MHC-I-opathies” [46].

In conclusion, data presented in this paper show that NC-B27 molecules are expressed in the joints and gastrointestinal tract in both human SpA and in the (21-3 × 283-2) F_1_ (B27 TG^1^) rat model of human SpA. These findings are consistent with the hypothesis that expression of non-conventional forms of B27 may amplify immune-mediated inflammation.

## Contributors

OR designed the experimental plan, acquired, analyzed and interpreted the data, and drafted the manuscript. She is a guarantor. KM, SK, DB, LvD, MvT, FC, GG provided key reagents or biological material, and acquired and analyzed data. KM, SK, FC, DB and PB were involved in conception and experimental design and manuscript writing and had final approval of the version to be published. FC, GG, DB and PB selected and enrolled the patients. OR and GG performed the experiments and analysis of the results. OR and GG performed the statistical analysis. DB and PB provided overall supervision. OR, SK, KM and PB wrote the paper.

## Funding

OR was supported by Arthritis Research UK grant no 19611, and by an EMBO travel award. This work was supported by the Oxford National Institute of Health Research (NIHR) Biomedical Research Center the Oxford NIHR Biomedical Research Unit (PB).

## Competing interests

None declared.

## Patient consent

Obtained.

## Ethics approval

Ethics approval Oxfordshire Research Ethics Committee (COREC/06/Q1606/139) for University of Oxford and the Local Ethics Committee of the Academic Medical Center for the University of Amsterdam. Ethical committee and the institutional review board of the University of Palermo.

## Provenance and peer review

Not commissioned; externally peer reviewed.
